# Case Report: Donor-derived early T-cell precursor acute lymphoblastic leukemia following an allogeneic hematopoietic stem cell transplantation for pediatric severe aplastic anemia

**DOI:** 10.3389/fped.2026.1932013

**Published:** 2026-09-10

**Authors:** Zhi Chen, Ming Sun, Zhuo Wang, Li Yang, Yu Du, Fang Tao, Wei Wang, Lan-Nan Zhang, Hao Xiong

**Affiliations:** Department of Hematology and Oncology, Wuhan Children’s Hospital, Tongji Medical College, Huazhong University of Science and Technology, Wuhan, China

**Keywords:** allo-HSCT, donor cell leukemia, ETP-ALL, severe aplastic anemia, umbilical cord blood

## Abstract

Donor cell leukemia is a rare but severe complication following an allogeneic hematopoietic stem cell transplantation (allo-HSCT), particularly in patients with non-malignant diseases, due to its aggressive clinical progression and poor prognosis. In recent years, with the increasing number of umbilical cord blood stem cell transplantation cases, umbilical cord blood–derived donor cell leukemia has garnered growing attention. Although umbilical cord blood cells may inherently carry tumor precursor characteristics, host environmental factors, immunosuppression, and chemotherapy during transplantation can exacerbate their transformation into leukemia. Herein, we report a clinical case of a pediatric patient with severe aplastic anemia who developed umbilical cord blood–derived early T-cell precursor acute lymphoblastic leukemia 2 years and 5 months after receiving an allo-HSCT. Despite multiple chemotherapy regimens, the patient ultimately failed to achieve remission and succumbed to the disease. Although this outcome is regrettable and highlights the refractory nature of post-transplant donor cell leukemia, further investigation into its molecular biological mechanisms is warranted to develop early prevention strategies and more effective therapeutic approaches.

## 1 Introduction

Donor cell leukemia (DCL) is a rare complication of an allogeneic hematopoietic stem cell transplantation (allo-HSCT), particularly when originating from cord blood hematopoietic stem cells. Approximately 20–30 cases of cord blood–derived DCL have been documented as of January 2026 (1). The reported overall incidence of DCL varies widely across studies, ranging from 0.12% to 5%. The recent widespread use of short tandem repeat (STR) chimerism analysis has enabled accurate and timely identification of the origins of normal or abnormal cells in patients after transplantation. Despite ongoing advancements in detection technologies, the observed incidence of DCL has not increased, suggesting that the rarity is not attributable to limitations in detection methods (2). In 1971, Fialkow et al. reported the first documented case of leukemic transformation of donor cells within the recipient following an allo-HSCT (3). The first reported case of cord blood-derived DCL was published in 2005 by Fraser (4). Herein, we report the first case of early T-cell precursor acute lymphoblastic leukemia (ETP-ALL) of cord blood derived after an allo-HSCT in a child with severe aplastic anemia (SAA); the leukemia cells were detected in the patient at 2 years and 5 months post-transplant. Exploring the potential onset and progression mechanisms enhances the understanding of the risks associated with cord blood use in clinical transplantation. In the present study, informed consent for the collection of all specimens and the performance of all diagnostic tests was obtained from the parents of the patient.

## 2 Case report

A 7-year 2-month-old girl was admitted on 12 April 2022, with a 1-week history of oral pain and bilateral lower-extremity petechiae, and a 3-day history of abnormal blood counts. A complete blood count revealed the following: white blood cell count, 3.98 × 10^9^/L; red blood cell count, 3.45 × 10^12^/L; hemoglobin, 97.0 g/L; platelet count, 17.0 × 10^9^/L; neutrophil count, 0.28 × 10^9^/L; and reticulocyte count, 18.3 × 10^9^/L. A further diagnostic workup (Table 1) confirmed the diagnosis of SAA.

**Table 1:** Hematologic parameters of the patient.

Item	SAA diagnosis (14 April 2022)	12 January 2025	8 March 2025	30 March 2025	30 April 2025	28 August 2025
Bone marrow morphology	A bone marrow examination revealed hypocellularity with decreased granulocytic and erythroid lineages. The lymphoid cells constituted 84% of nucleated cells. Megakaryocytes were absent on the smear. Two fatty marrow particles were noted.	The bone marrow was hypercellular. Lymphocytes accounted for 49% of all nucleated cells, with blasts and immature lymphocytes comprising 25% of the total nucleated cells.	The marrow was hypercellular with rare erythroid lineages. Blasts accounted for approximately 29% of cells, exhibiting variable cell size, some irregular shapes, scant to moderate cytoplasm, and occasional cytoplasmic tails. Nuclear chromatin was relatively fine, and nucleoli were variably prominent. A few megakaryocytes were observed.	The bone marrow was hypercellular, with the suppression of both granulocytic and erythroid lineages. Blasts accounted for approximately 67%, displaying variable, predominantly large cell size, abundant cytoplasm, and occasional azurophilic granules. Nuclear chromatin was relatively fine, nuclei were irregularly shaped, with occasional twisting and folding. Megakaryocytes were scarce throughout the smear. Cytochemical staining demonstrated <3% peroxidase-positive cells, fine periodic acid-Schiff-positive granules, and negative reactions for both alpha-naphthyl acetate esterase and chloroacetate esterase, suggestive of acute myeloid leukemia, with the M5 subtype not excluded.	The bone marrow was hypocellular, with the suppression of both granulocytic and erythroid lineages, and megakaryocytes were scarcely observed throughout the smear. Blasts accounted for approximately 10% of cells, exhibiting variable cell size, abundant cytoplasm, occasional azurophilic granules, fine nuclear chromatin, irregular nuclear shapes with occasional twisting and folding, and variably prominent nucleoli.	The bone marrow was hypercellular, with blasts comprising approximately 30%. The blasts exhibited variable cell size, abundant cytoplasm, and occasional azurophilic granules. Nuclear chromatin was fine, nuclei were partially irregular in shape with occasional twisting or folding, and nucleoli were variably prominent. No megakaryocytes were observed throughout the smear.
Bone marrow biopsy	A bone marrow biopsy demonstrated hypocellularity with overall reduced hematopoiesis. Notably, focal areas had granulocytic and erythroid hyperplasia. Megakaryocytes were markedly decreased, with only two identified on the smear. No significant fibrosis was observed.	—				
Immunophenotyping	The CD34^+^/CD117^+^ cell population was sparse, with a relatively increased proportion of lymphocytes. No immunophenotypically abnormal B- or T-lymphocyte populations were identified, and the CD4/CD8 ratio was approximately 0.97. The proportion of granulocytes was relatively low and consisted predominantly of mature granulocytes, with no immunophenotypically abnormal immature myeloid cell population detected.	An abnormal cell population accounting for approximately 13.4% was identified, predominantly expressing CD3, CD7, CD4, CD34, CD38, CD45, CD56, CD117, and HLA-DR, while lacking TDT, CD1a, CD2, CD3, CD5, CD8, CD10, CD11b, CD11c, C13, CD14, CD15, CD16, CD19, CD20, CD22, CD33, CD64, IPO, CD79a, TCR*αβ*, TCR*γ*, Kappa, and Lambda expression.	The proportion of suspicious abnormal cells was 13.20%.	Approximately 25% of cells primarily expressed CD11c, CD33, CD38, CD64, and HLA-DR, with a partial expression of CD4, CD15, and CD117 (low levels), and lacking the expression of CD2, CD3, CD5, CD7, CD8, CD10, CD13, CD14, CD19, CD20, CD22, CD34, CD36, CD56, cCD3, cCD79a, MPO, and TDT, suggestive of abnormal immature monocytes; approximately 4.5% of cells expressed CD7, CD56, CD11c, CD33, CD38, CD34, CD64, and HLA-DR, with a partial expression of CD4 and CD117 (low levels), and lacking the expression of CD2, CD3, CD5, and CD8.	Approximately 0.7% abnormal cells were detected, primarily expressing CD34, CD56, CD7, and CD11c, with a partial expression of CD117 and CD4, and lacking the expression of CD14 and CD64; in addition, an abnormal myeloid cell population comprising approximately 16.2% of cells was observed.	The proportion of suspicious abnormal cells was 32.0%.
Genetic mutations in myelodysplastic syndrome	Negative.	—				
Chromosome karyotype analysis	No abnormal mitotic figures were observed.	45, XY, −7[12]/46, XY, idem, +mar[6]/46, XY[2].				
Bone marrow imaging	Reduced systemic bone marrow activity was observed, with no evident signs of peripheral bone marrow expansion.	—				
Gene sequencing	No congenital bone marrow failure or immunodeficiency pathogenic genes were detected.	Whole-transcriptome sequencing revealed mutations in *NRAS* (Q61 K), *JAK2* (T875N), and *ETV6* (R105X).				
Paroxysmal nocturnal hemoglobinuria tests	CD55 and CD59 expression levels were within normal ranges.	—				

Following the diagnosis, the patient received oral cyclosporine therapy. In accordance with the *Guidelines for the Diagnosis and Treatment of Pediatric Aplastic Anemia (2019 Edition)*, the patient met clear indications for an allo-HSCT. As no human leukocyte antigen (HLA)-matched sibling donor was available, an unrelated donor from the Chinese Marrow Donor Program was identified with an HLA match of 11/12. After a thorough discussion of the disease prognosis and treatment options with the patient's parents, they agreed to proceed with an unrelated donor allo-HSCT. In July 2022, the patient was admitted to the transplant ward and received conditioning chemotherapy with a regimen of busulfan, cyclophosphamide, and antithymocyte globulin [cyclophosphamide, 60 mg/kg per day, IV, 2 days (−8 to −7 days); fludarabine, 40 mg/m^2^ per day, IV, 5 days (−6 to −2 days); busulfan, 1.1 mg/kg per dose, Q6H, IV (−6 to −5 days); and antithymocyte globulin (7.5 mg/kg) for 3 consecutive days starting on −4 to −2 days]. On 8 August 2022, third-party umbilical cord blood stem cells (provided by the Shandong Cord Blood Bank, donor ID A0123416, male, 10/10 HLA match, blood type B Rh^+^) were infused, delivering a nucleated cell dose of 3.87 × 10^7^/kg and a CD34^+^ cell dose of 1.52 × 10^5^/kg. The following day (9 August 2022), the patient was infused with peripheral blood stem cells from an unrelated donor (11/12 HLA match, blood type B Rh^+^) sourced from the Chinese Marrow Donor Program, providing a mononuclear cell dose of 6.7 × 10^8^/kg and a CD34^+^ cell dose of 6.6 × 10^6^/kg. The graft-vs.-host disease (GVHD) prophylaxis regimen consisted of cyclosporine with mycophenolate mofetil and low-dose methotrexate. Cyclosporine A (CSA) was started at 1.5 mg/kg per day IV (−9 to −2 days) and was changed to 3 mg/kg day on day 1 prior to HSCT. The CSA levels were monitored twice a week (therapeutic range, 100–250 ng/mL). Mycophenolate mofetil was started at 30 mg/kg daily (−9 to +30 days), and then the dose was reduced to half from +31 to +45 days. Methotrexate was administered on day 1 (15 mg/m^2^), day 3 (10 mg/m^2^), and day 6 (10 mg/m^2^) after the transplant.

**Figure 1:**
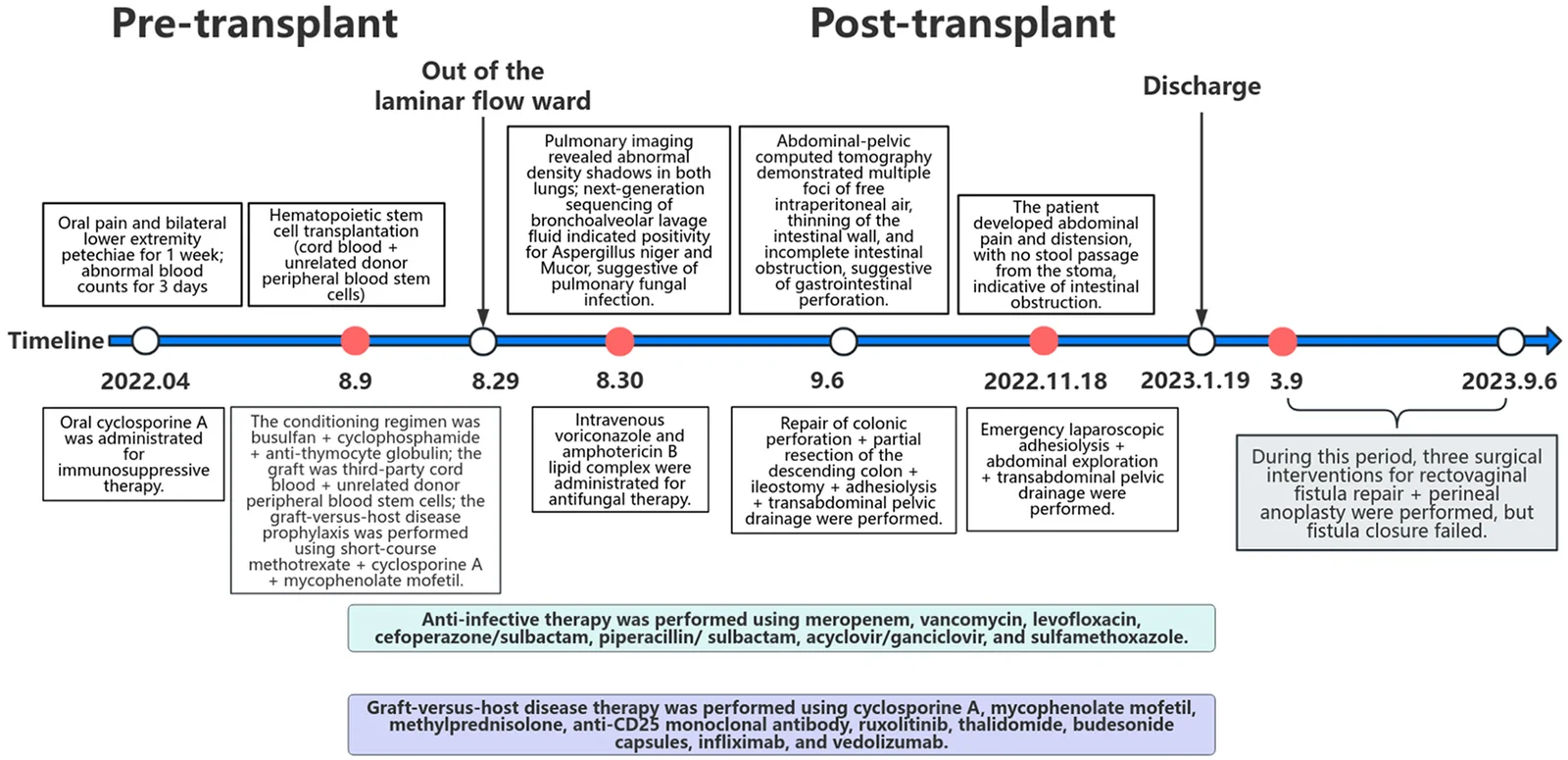
A timeline of key clinical events and management strategies from April 2022 to September 2023. Major events include symptoms (e.g., chief complaint, pulmonary fungal infection, gastrointestinal perforation, abdominal pain, and abdominal distension), complications (e.g., pneumonia and intestinal GVHD), and treatment milestones (e.g., immunosuppressive therapy, HSCT, anti-infective therapy, and surgical interventions). Significant events, such as transplantation and surgical procedures, are annotated specifically.

On day +10 after stem cell infusion, the patient developed hematochezia, hematemesis, and other symptoms, which were considered gastrointestinal GVHD. Pulmonary aspergillosis occurred on day +21. After the administration of anti-GVHD and antifungal therapies, the clinical symptoms gradually alleviated. The patient was transferred from the laminar flow ward for subsequent treatment on 29 August. One month postinfusion, the girl developed gastrointestinal perforation. An emergency surgery was performed, combined with active anti-infection, anti-GVHD treatment, and supportive care including parenteral nutrition. More than 5 months after the transplantation, the patient was discharged with an ostomy bag in place. Because of a rectovaginal fistula, multiple surgical repairs were conducted, yet fistula closure could not be achieved. Figure 1 illustrates the detailed treatment course.

Routine peripheral blood chimerism monitoring was performed following the allo-HSCT. On 22 August (day +13), a STR analysis of peripheral blood revealed that the proportion of cells from the unrelated donor was 99.70%. Competitive engraftment of cord blood cells was detected at day +40 post-transplant (19 September 2022), accounting for 10.72%, while cells derived from the unrelated donor accounted for 89.28%. Thereafter, chimerism was assessed approximately every 2 weeks. The proportion of cord blood–derived cells progressively increased over time. By 2 March 2024 (1 year and 7 months post-transplant), a repeat chimerism evaluation demonstrated 83.06% cord blood–derived cells, confirming mixed chimerism involving both cord blood and unrelated donor cells.

**Figure 2:**
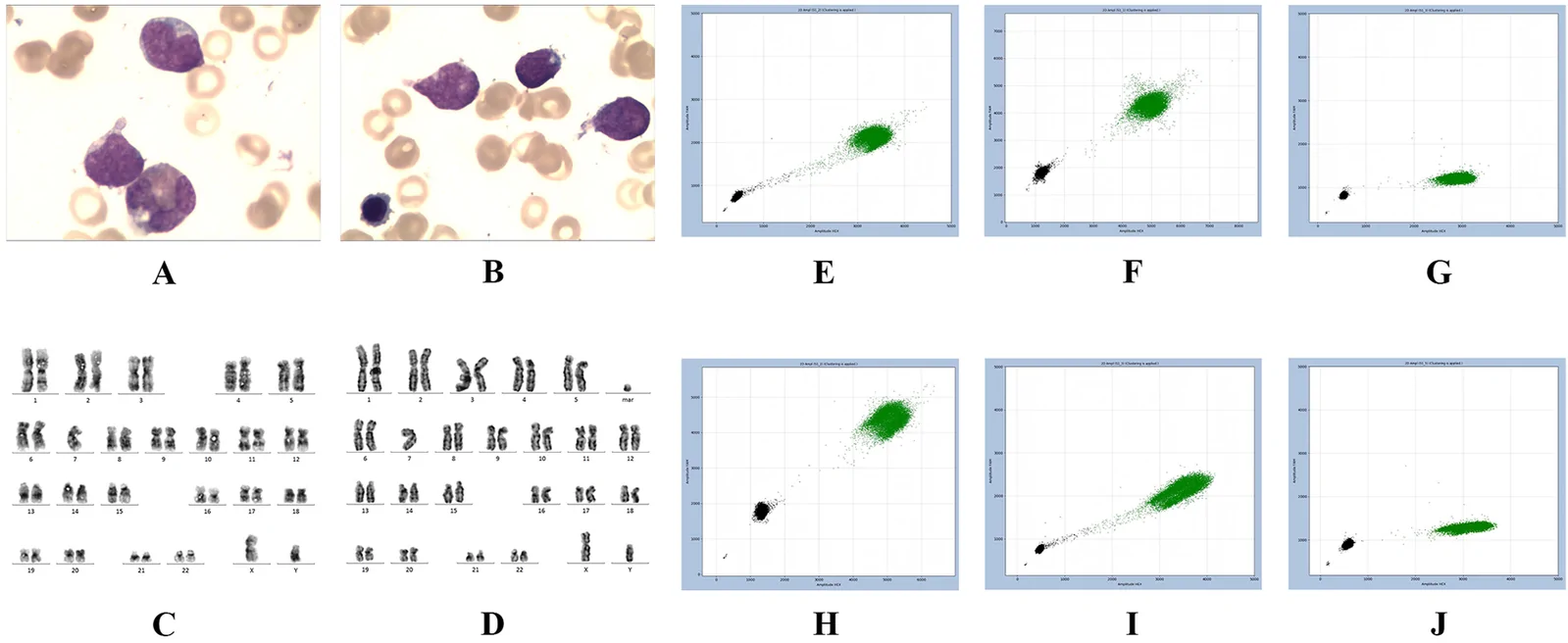
Morphological, cytogenetic characteristics, and molecular biology test results of the patient with acute leukemia. **(A,B)** Morphological features of bone marrow cells at the time of ETP-ALL diagnosis; **(C,D)** A Karyotype analysis of bone marrow cells at the time of ETP-ALL diagnosis; **(E)** NRAS (Q61K) mutation was negative in cord blood stem cells; **(F)** JAK2 (T875N) mutation was negative in cord blood stem cells; **(G)** ETV6 (R105X) mutation was negative in cord blood stem cells; **(H)** NRAS (Q61K) mutation was negative in unrelated donor stem cells; **(I)** JAK2 (T875N) mutation was negative in unrelated donor stem cells; **(J)** ETV6 (R105X) mutation was negative in unrelated donor stem cells; The upper-left data points represent mutation-positive cells; the upper-right data points represent mutation-positive and wild-type wells; the lower-right data points represent wild-type wells; the lower-left data points represent no-target wells.

On 12 January 2025 (2 years and 5 months post-transplant), the patient presented with a 3-day history of headache. The complete blood count revealed the following: white blood cell count, 4.2 × 10^9^/L; hemoglobin, 74 g/L; platelet count, 49 × 10^9^/L; and neutrophil count, 1.22 × 10^9^/L. A peripheral blood morphology assessment revealed cells with fine chromatin. A cerebrospinal fluid analysis, including biochemical, routine, and microbiological examinations, revealed no abnormalities. Electroencephalography and non-contrast brain magnetic resonance imaging revealed no significant abnormalities. A bone marrow chimerism analysis indicated 92.91% cord blood donor cells and only 7.09% unrelated donor cells. A bone marrow cytological analysis revealed a lymphocyte proportion of 49%, within which blasts and immature lymphocytes accounted for 25%. These cells exhibited variable size, scant basophilic cytoplasm, irregular nuclear contours with notches or indentations in a subset, relatively uniform and fine chromatin, and nucleoli of variable prominence (Figures 2A,B). Flow cytometric immunophenotyping of the bone marrow cells demonstrated the expression of cytoplasmic CD3 (55.7%), CD7 (68.6%), and CD4 (23.9%). The identified stem cell markers included CD34 (47.7%), CD117 (17.9%), and HLA-DR (81.4%). The T-cell markers CD8 and CD1a were negative, with weak CD5 expression (5.9%). Table 1 details the bone marrow findings. The patient was diagnosed with ETP-ALL based on established diagnostic criteria and scoring systems (5, 6).

The sources of the allo-HSCT for the patient included an unrelated donor and third-party cord blood; both donors were male- and sex-mismatched with the patient. A chromosomal karyotype analysis revealed that all the tested cells displayed male karyotypes. Of the 20 metaphase cells analyzed, 18 exhibited abnormal karyotypes: 12 cells showed monosomy 7 (Figure 2C), and the remaining 6 additionally harbored an extra marker chromosome (Figure 2D). The karyotype was described as 45, XY, −7[12]/46, idem, +mar[6]/46, XY[2]. The presence of the Y chromosome across all analyzed cells confirmed that the leukemic cells were of donor origin. However, the cytogenetic analysis alone could not distinguish between the unrelated donor and the third-party cord blood donor, necessitating further examinations.

**Figure 3:**
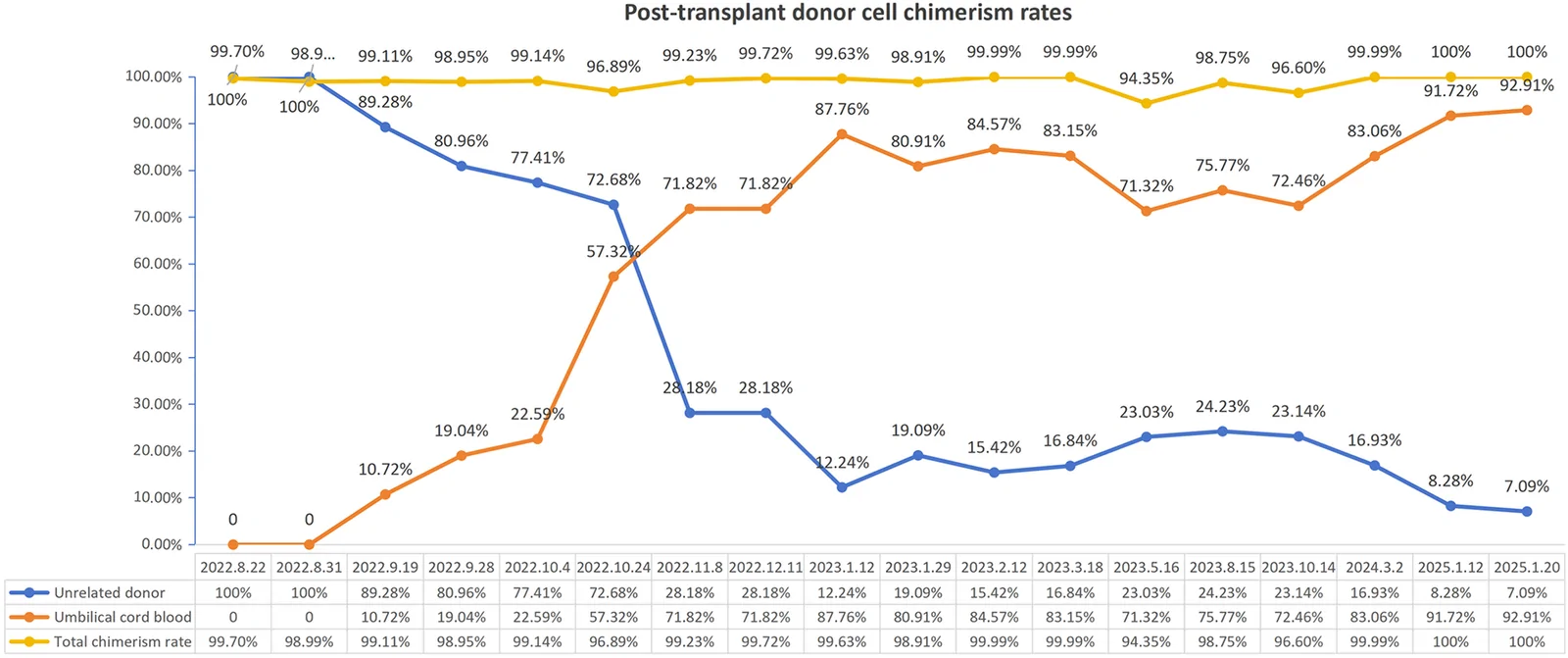
Dynamic changes in the chimerism rates of donor-derived cells in the patient post-transplant.

The chimerism analysis was primarily performed using short tandem repeat genotyping, which is a highly effective method for definitively establishing the origin of leukemia (7). Donor-derived cells accounted for 99.11% at day +40 post-transplant, indicating complete donor chimerism. Subsequent serial monitoring of chimerism using the same method targeting 25 genetic loci consistently demonstrated mixed chimerism involving both cord blood cells and unrelated donor cells (Figure 3). On 20 January 2025 (2 years and 5 months post-transplant), a chimerism analysis of a bone marrow sample revealed that 92.91% cord blood donor cells were present, whereas only 7.09% unrelated donor cells were present. Given the coexistence of cells from two distinct donor sources within the patient, CD34^+^ cells were isolated from a concurrently collected peripheral blood sample using immunomagnetic bead selection. The chimerism analysis was repeated, which demonstrated 100% cord blood donor origin and 0% unrelated donor origin, indicating complete cord blood donor chimerism. These findings conclusively established that the leukemic cells originated from the cord blood hematopoietic stem cells.

An examination of the patient's bone marrow samples before transplantation did not help identify any significant tumor-associated gene mutations. Following transplantation, and upon the onset of leukemia, high-throughput sequencing was done to detect single-nucleotide variants and insertions/deletions. Both hematologic tumor whole-transcriptome sequencing analysis and hematologic tumor gene mutation detection identified the mutation sites *NRAS* (Q61K), *JAK2* (T875N), and *ETV6* (R105X). In several reports, these gene mutations have been clearly associated with acute leukemia in clinical practice (8–10). The *NRAS* (Q61K), *JAK2* (T875N), and *ETV6* (R105X) mutation ratios were quantified using a digital PCR system. The results indicated that all aforementioned gene mutations were negative in both the cord blood and unrelated donor hematopoietic stem cells (Figures 2E–J).

Following the diagnosis, the parents declined the performance of intensive chemotherapy for their patient and instead opted for oral venetoclax (25 mg/day on the first day, followed by 50 mg/day thereafter, administered at a fixed time daily) combined with chidamide tablets (10 mg/day, administered orally twice weekly). After 3 weeks of treatment (8 March 2025), a repeat bone marrow evaluation revealed no remission (Table 1). The parents then consented to chemotherapy for their patient, following which induction chemotherapy was administered according to the CCLG-ALL2018 protocol using a VDLD regimen (vincristine + daunorubicin + L-asparaginase + dexamethasone). A reassessment after 3 weeks (30 March 2025) revealed unexpected findings: bone marrow morphology raised the suspicion of acute myeloid leukemia (AML). Flow cytometric immunophenotyping suggested the expression of myeloid markers in the leukemic cells (Table 1). Therefore, treatment was adjusted to the VAH regimen (venetoclax + cytarabine + homoharringtonine) according to the CCLG-AML2024 protocol.

The treatment response was reassessed 3 weeks later (30 April 2025). The bone marrow examination results indicated persistent disease despite multiple courses of chemotherapy; the specific situation is presented in Table 1. Given the treatment refractoriness, a salvage second HSCT was advised; however, the parents declined and opted to continue with chemotherapy. Considering the potentially poor tolerance to intensive chemotherapy, the subsequent 2 months involved hypomethylating agents (azacitidine/decitabine) combined with venetoclax (50 mg/dose) administered once weekly. In addition, given the presence of a *JAK2* mutation, oral ruxolitinib (5 mg/dose, once daily) was added to the treatment plan. On 2 July 2025, a bone marrow evaluation revealed a slightly hypocellular marrow with 18% blasts morphologically and 17.8% abnormal cells by a flow cytometry minimal residual disease analysis, confirming ongoing progression and poor response to chemotherapy. The poor prognosis was reiterated to the parents. On 12 July 2025, the parents consented to a reduced-intensity FLAG regimen (fludarabine + cytarabine + granulocyte colony-stimulating factor). After hematopoietic recovery from myelosuppression, a bone marrow assessment on 28 August 2025 revealed 30% blasts morphologically and 32% abnormal cells by a flow cytometry minimal residual disease analysis. Because of the persistently poor response to chemotherapy, the parents declined further active treatment for their patient and asked for the girl to be shifted to palliative and supportive care. However, unfortunately, the patient died on 15 October 2025 (9 months after the leukemia diagnosis) because of progressive leukemia complicated by disseminated intravascular coagulation and intracranial hemorrhage.

## 3 Discussion

Limited publicly reported case data on leukemia in donor cells after transplantation are available for research, and the conditions of HSCT are highly heterogeneous. Consequently, elucidating the pathogenesis of DCL is challenging. A current theory posits that hematopoietic stem cells may sustain a “first hit” in the donor (e.g., genotype instability or inherited defects in regulatory genes), followed by a “second hit” in the aberrant host environment (e.g., the toxic effects of pretransplant conditioning regimens with chemotherapy and radiotherapy, or a disruption of the host bone marrow microenvironment) (11). This sequence of events may promote the malignant proliferation of donor-derived stem cells. However, this is merely a hypothesis based on the current observed phenomena. Furthermore, the post-transplant administration of immunosuppressive agents, including corticosteroids and cyclosporine, diminishes recipient immune surveillance, which impairs the ability of the immune system to rapidly recognize and eliminate clonal evolution in engrafted donor cells, thereby increasing the risk of leukemic transformation (12). In our patient, 1 year after transplantation, a comprehensive evaluation revealed no significant active GVHD. The immunosuppressive agent (CSA) was therefore gradually tapered, and cyclosporine was discontinued in September 2023 to facilitate the prompt restoration of the child's immune function. Subsequently, regular monitoring of immune cell recovery was performed, and the results indicated that both CD4+ and CD8+ T-cell counts had returned to normal levels. As of January 2025, when the child was diagnosed with leukemia, she had undergone preparative chemotherapy 28 months prior and had been off immunosuppressive therapy for nearly 15 months; regular monitoring of peripheral blood cell counts and morphology after discontinuation of CSA revealed no abnormalities. Therefore, it is not advisable to directly attribute the development of donor cell leukemia to pretreatment chemotherapy or immunosuppressants; rather, it is more reasonable to infer that these factors played a contributory and catalytic role in this patient.

Our patient was initially diagnosed with SAA rather than a malignant hematological disorder. No leukemia-associated molecular abnormalities were detected in the patient prior to transplantation, and subsequent verification of the graft revealed no relevant genetic anomalies, indicating that neither the recipient nor the graft harbored leukemia-promoting genes before transplantation. However, the patient developed DCL following transplantation, with *NRAS* (Q61K), *JAK2* (T875N), and *ETV6* (R105X) mutations identified in the bone marrow, which were confirmed to have originated from the cord blood. These findings suggested that the cord blood stem cells underwent genetic mutations within the unique hematopoietic microenvironment after engraftment in the patient, progressing to leukemic cells.

The bone marrow stroma is pivotal in hematopoietic reconstitution (13), particularly in patients undergoing an allo-HSCT, where donor cells rely on host-derived bone marrow stromal cells for proliferation and differentiation. A previous report documented a patient who developed two clones of donor-derived acute myeloid leukemia following a sequential allo-HSCT from both a related donor and an unrelated donor (14), providing evidence of the important influence of the host microenvironment on leukemogenesis. In our patient, follow-up assessments of both the unrelated donor and the cord blood donor, including first-degree relatives of the umbilical cord blood donor, revealed good health status in both individuals, indicating that distinct microenvironments affect genetically identical stem cells differently. Regrettably, biomarkers and methodologies for the direct detection of defects in the bone marrow stromal microenvironment remain lacking, rendering it challenging to fully elucidate the underlying mechanisms (3). The relevant mechanisms require further validation through basic research.

Our patient was diagnosed with unrelated cord blood–derived ETP-ALL following an allo-HSCT. ETP-ALL is a subtype of acute T lymphoblastic leukemia characterized by the migration of T-cell precursors at the early differentiation stage from the bone marrow to the thymus. This cellular population expresses a diverse range of T-lineage, stem cell, and myeloid-associated transcripts, retains multipotent differentiation potential, and represents a poorly differentiated, stem cell–like leukemia characterized by maturation arrest (15). ETP-ALL has been indicated to lack distinctive clinical manifestations, with bone marrow cells exhibiting no typical morphological or pathological features. However, ETP-ALL demonstrates unique immunophenotypic characteristics (16), including the expression of stem cell antigens (e.g., CD34 and HLA-DR) or myeloid antigens (e.g., CD11b, CD13, and CD117). Consequently, diagnosis primarily relies on immunophenotyping. To date, no ETP-ALL cases arising from cord blood cells following an allo-HSCT have been reported, and no standardized therapeutic approach has been established in clinical practice.

Our patient was initially treated according to the standard T-ALL protocol using the classic VDLD regimen for induction chemotherapy; however, leukemia remission was not achieved.

Subsequent bone marrow immunophenotyping revealed a shift toward the myeloid lineage, with the expression of markers such as CD11c, CD33, CD38, CD64, and HLA-DR. Notably, markers CD33, CD11c, and CD64 were not detected at the time of initial ETP-ALL diagnosis, suggesting that these myeloid precursor cells are newly generated. They likely originate from a single clonal progenitor cell, expand gradually under the influence of the microenvironment, and ultimately progress to AML. The COG Pediatric ETP-ALL guideline indicates that ETP-ALL cells have myeloid differentiation potential, and lineage conversion can occur after treatment. Thus, once the disease progresses to AML, it should be managed as high-risk AML, and we promptly adjusted the chemotherapy regimen. Given the similarities in cellular origin and genetic profile between ETP-ALL and myeloid leukemia, acute myeloid leukemia treatment regimens incorporating high-dose cytarabine were recommended (17, 18). Accordingly, we implemented therapeutic strategies for acute myeloid leukemia, including intensive regimens such as VAH and FLAG, and less intensive options such as decitabine combined with venetoclax; nevertheless, the patient failed to achieve remission. Although a salvage second transplantation from an alternative donor is a potential option discussed in the literature (19), this approach is associated with high treatment-related mortality. In our patient, after considering both the therapeutic risks and their financial implications, and given the persistent failure to achieve remission despite multiple chemotherapy regimens, the patient’s parents chose to pursue palliative care for their child until her unfortunate death.

Although current technological capabilities enable the screening of potential clonal hematopoiesis in donors, the predictive utility of such assessments remains inconclusive, while the substantial economic costs limit their widespread adoption (15). Several enhancements can be implemented to mitigate the medical risks associated with DCL following HSCT. First, transplant physicians should explicitly inform patients about the possibility of donor-derived hematopoietic malignancies. Despite their exceedingly low incidence, patients retain the right to be apprised of this risk. Second, both donors and recipients should be encouraged to promptly report post-transplant health status to the donor registry system, and periodic follow-up by the registry is particularly important. Finally, should such complications arise, clinicians must prioritize early recognition, especially when clinical manifestations diverge from those of the primary disease, and consider graft-derived complications in the differential diagnosis. Ultimately, advancing the investigation of DCL pathogenesis, encompassing both clinical and fundamental research endeavors, requires the use of large-scale, multicenter datasets. This approach would facilitate the identification of common features among pertinent cases and enable a thorough analysis, representing a key objective for our future investigations.

## 4 Conclusion

In conclusion, we report the first case of umbilical cord blood–derived ETP-ALL occurring in a pediatric patient with aplastic anemia following an allo-HSCT. The patient's subsequent unfortunate death underscores the necessity to identify the origin of malignant cells as early as possible upon detection after an allo-HSCT, enabling more accurate prognosis assessment and tailored treatment planning. Furthermore, although the likelihood of non-consanguineous cord blood cells harboring malignant cells is negligible, clinical evidence suggests that umbilical cord blood banks should conduct long-term health follow-ups of donors to facilitate early identification of potential malignant defects in donor cells.

## Data Availability

The raw data supporting the conclusions of this article will be made available by the authors, without undue reservation.
