# Supplementary material for: Case Report: Donor-derived early T-cell precursor acute lymphoblastic leukemia following an allogeneic hematopoietic stem cell transplantation for pediatric severe aplastic anemia
Source: Front Pediatr. 2026 Sep 10;14:1932013. doi: 10.3389/fped.2026.1932013 (PMC13601031; doi:10.3389/fped.2026.1932013)
Supplement: Supplementary file 1 [file Table1.docx]

***Supplementary Material***

**Table 1. Hematologic Parameters of the Patient.**

| **Item** | **SAAdiagnosis（April14,2022）** | **January12,2025** | **March8,2025** | **March30,2025** | **April30,2025** | **August28,2025** |
| --- | --- | --- | --- | --- | --- | --- |
| **Bone marrow morphology** | Bone marrow examination revealed hypocellularity with decreased granulocytic and erythroid lineages. The lymphoid cells constituted 84% of nucleated cells. Megakaryocytes were absent on the smear. Two fatty marrow particles were noted. | The bone marrow was hypercellular. Lymphocytes accounted for 49% of all nucleated cells, with blasts and immature lymphocytes comprising 25% of the total nucleated cells. | Marrow was hypercellular with rare erythroid lineages. Blasts accounted for approximately 29% of cells, exhibiting variable cell size, some irregular shapes, scant to moderate cytoplasm, and occasional cytoplasmic tails. Nuclear chromatin was relatively fine, and nucleoli were variably prominent. A few megakaryocytes were observed. | The bone marrow was hypercellular, with suppression of both granulocytic and erythroid lineages. Blasts accounted for approximately 67%, displaying variable, predominantly large cell size, abundant cytoplasm, and occasional azurophilic granules. Nuclear chromatin was relatively fine, nuclei were irregularly shaped, with occasional twisting and folding. Megakaryocytes were scarce throughout the smear. Cytochemical staining demonstrated <3% peroxidase-positive cells, fine periodic acid-Schiff-positive granules, and negative reactions for both alpha-naphthyl acetate esterase and chloroacetate esterase, suggestive of acute myeloid leukemia, with the M5 subtype not excluded. | Bone marrow was hypocellular, with suppression of both granulocytic and erythroid lineages, and megakaryocytes were scarcely observed throughout the smear. Blasts accounted for approximately 10% of cells, exhibiting variable cell size, abundant cytoplasm, occasional azurophilic granules, fine nuclear chromatin, irregular nuclear shapes with occasional twisting and folding, and variably prominent nucleoli. | Bone marrow was hypercellular, with blasts comprising approximately 30%. The blasts exhibited variable cell size, abundant cytoplasm, and occasional azurophilic granules. Nuclear chromatin was fine, nuclei were partially irregular in shape with occasional twisting or folding, and nucleoli were variably prominent. No megakaryocytes were observed throughout the smear. |
| **Bone marrow biopsy** | Bone marrow biopsy demonstrated hypocellularity with overall reduced hematopoiesis. Notably, focal areas had granulocytic and erythroid hyperplasia. Megakaryocytes were markedly decreased, with only two identified on the smear. No significant fibrosis was observed. | - |  |  |  |  |
| **Immunophenotyping** | The CD34^+^/CD117^+^ cell population was sparse, with a relatively increased proportion of lymphocytes. No immunophenotypically abnormal B- or T-lymphocyte populations were identified, and the CD4/CD8 ratio was approximately 0.97. The proportion of granulocytes was relatively low and consisted predominantly of mature granulocytes, with no immunophenotypically abnormal immature myeloid cell population detected. | An abnormal cell population accounting for approximately 13.4% was identified, predominantly expressing CD3, CD7, CD4, CD34, CD38, CD45, CD56, CD117, and HLA-DR, while lacking TDT, CD1a, CD2, CD3, CD5, CD8, CD10, CD11b, CD11c, C13, CD14, CD15, CD16, CD19, CD20, CD22, CD33, CD64, IPO, CD79a, TCRαβ, TCRγ, Kappa, and Lambda expression. | The proportion of suspicious abnormal cells was 13.20%. | Approximately 25% of cells primarily expressed CD11c, CD33, CD38, CD64, and HLA-DR, with partial expression of CD4, CD15, and CD117 (low levels), lacking expression of CD2、CD3、CD5、CD7、CD8、CD10、CD13、CD14、CD19、CD20、CD22、CD34、CD36、CD56、cCD3、cCD79a、MPO、TDT, suggestive of abnormal immature monocytes;approximately 4.5% of cells expressed CD7, CD56, CD11c, CD33, CD38, CD34, CD64, and HLA-DR,with partial expression of CD4,CD117(low levels),and lacking expression of CD2、CD3、CD5、CD8. | Approximately 0.7% abnormal cells were detected, primarily expressing CD34, CD56, CD7, and CD11c, with partial expression of CD117 and CD4, and lacking expression of CD14 and CD64; additionally, an abnormal myeloid cell population comprising approximately 16.2% of cells was observed. | The proportion of suspicious abnormal cells was 32.0%. |
| **Genetic mutations in myelodysplastic syndrome** | Negative. | - |  |  |  |  |
| **Chromosome karyotype analysis** | No abnormal mitotic figures were observed. | 45, XY, -7[12]/46, XY, idem, +mar[6]/46, XY[2]. |  |  |  |  |
| **Bone marrow imaging** | Reduced systemic bone marrow activity was observed, with no evident signs of peripheral bone marrow expansion. | - |  |  |  |  |
| **Gene sequencing** | No congenital bone marrow failure or immunodeficiency pathogenic genes were detected. | Whole-transcriptome sequencing revealed mutations in *NRAS* (Q61K), *JAK2* (T875N), and *ETV6* (R105X). |  |  |  |  |
| **Paroxysmal nocturnal hemoglobinuria tests** | CD55 and CD59 expression levels were within normal ranges. | - |  |  |  |  |
